# Protocol to generate stable knockout lines in the human-parasitic nematode *Strongyloides stercoralis*

**DOI:** 10.1016/j.xpro.2025.104201

**Published:** 2025-11-14

**Authors:** Navonil Banerjee, Breanna Walsh, Ruhi Patel, Michelle L. Castelletto, Elissa A. Hallem

**Affiliations:** 1 Department of Microbiology, Immunology, and Molecular Genetics, University of California, Los Angeles, Los Angeles, CA 90095, USA; 2 Molecular Biology Interdepartmental PhD Program, University of California, Los Angeles, Los Angeles, CA 90095, USA; 3 UCLA-Caltech Medical Scientist Training Program, University of California, Los Angeles, Los Angeles, CA, USA; 4 Molecular Biology Institute, University of California, Los Angeles, Los Angeles, CA 90095, USA

**Keywords:** CRISPR, Genetics, Microbiology, Model Organisms

## Abstract

A major limitation to the study of gene function in parasitic nematodes was the inability to make stable mutant lines. Here, we present a protocol for generating stable knockout lines in the human-parasitic nematode *Strongyloides stercoralis*. We describe steps for generating CRISPR components and microinjecting them into worms. We also detail procedures for identifying potential gene disruptions and propagating mutants by host passage in gerbils to generate stable homozygous knockout lines. This protocol enables studies of gene function in *S. stercoralis*.

For complete details on the use and execution of this protocol, please refer to Banerjee et al.^1^

## Highlights

• Protocol for generating stable mutant lines of *S. stercoralis* using CRISPR
• Steps for generating CRISPR constructs and microinjecting into free-living adults
• Instructions for infecting hosts with activated F_1_ iL3 progeny
• Guidance on isolation of F_3_ homozygous knockout iL3s and stable line maintenance

## Abstract (editor highlights)

Publisher’s note: Undertaking any experimental protocol requires adherence to local institutional guidelines for laboratory safety and ethics.

## Graphical abstract

**Figure:**
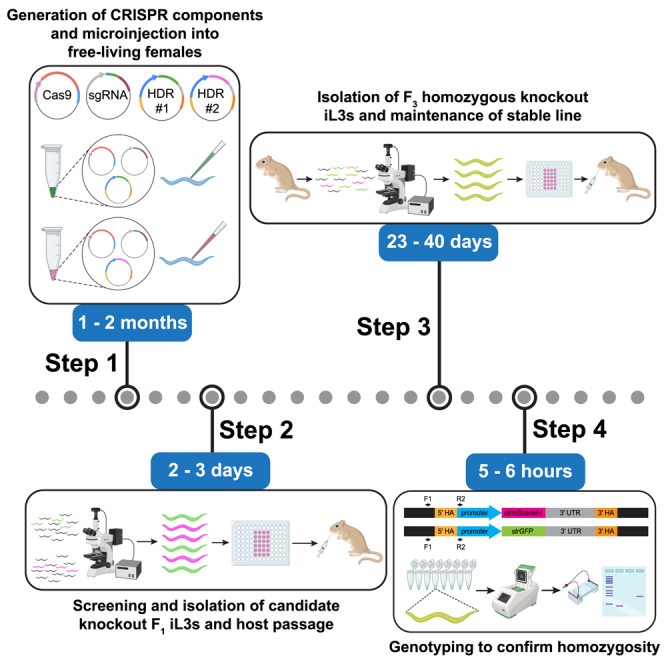

## Before you begin

The protocol below describes the steps necessary to obtain stable mutant lines in the human-parasitic, skin-penetrating nematode *Strongyloides stercoralis*. As a proof-of-principle, we focus on the gene encoding the receptor guanylate cyclase *Sst-*GCY-9, which is required for the detection of carbon dioxide (CO_2_), an important host-associated cue for *S. stercoralis* and other parasitic nematodes.^1^^,^^2^

**Figure 1:**
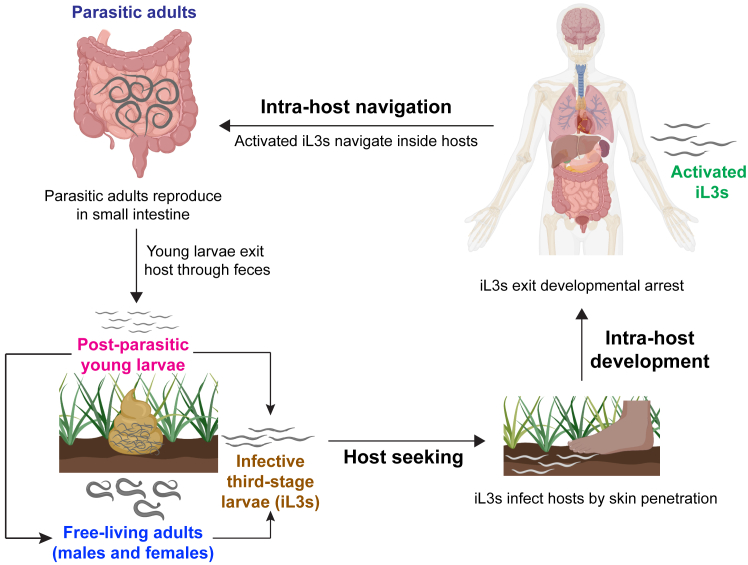
The life cycle of *S. stercoralis* The parasitic adults reproduce in the host small intestine and the progeny exit through host feces as post-parasitic, first-stage larvae. A subset of these larvae develops into developmentally arrested infective third-stage larvae (iL3s), while the remaining subset develops into a single generation of free-living adults. These free-living adults can be microinjected with exogenous DNA to generate transgenics or knockouts. All progeny of the free-living adults become iL3s. The iL3s invade hosts by skin penetration and resume development as activated iL3s to complete their parasitic life cycle, ending up as parasitic adults in the host small intestine. Worms are not to scale. Figure adapted from Banerjee et al., 2025.^1^

The historic lack of genetic tools in parasitic nematodes has largely prevented *in vivo* mechanistic studies of their basic biology. *S. stercoralis* is uniquely amenable to genetic manipulation because its life cycle includes a single free-living generation^3^^,^^4^ (Figure 1). Exogenous DNA can be introduced into the free-living adult females of *S. stercoralis* by intragonadal microinjection using techniques adapted from the free-living nematode *Caenorhabditis elegans*.^3^^,^^4^ In recent years, the development of a molecular genetic toolkit for *S. stercoralis*, including the generation of stable transgenic *S. stercoralis* lines,^5^ has established this parasite as a model to explore mechanisms of host-parasite interactions at the cellular and molecular level.^3^^,^^4^

Previous studies have described an approach for CRISPR/Cas9-mediated targeted mutagenesis in *S. stercoralis*.^6^^,^^7^^,^^8^^,^^9^^,^^10^^,^^11^^,^^12^ While this CRISPR pipeline greatly facilitated studies of the genetic mechanisms that regulate the behavior and physiology of these nematodes, it was limited to the generation of mutant F_1_ infective third-stage larvae (iL3s). This restricted phenotypic studies to F_1_ iL3s and precluded analyses of other life stages. Moreover, using this CRISPR pipeline, phenotypic assays had to be performed with single F_1_ iL3s of unknown genotype, followed by *post hoc* genotyping to identify iL3s with homozygous gene disruptions. Since only a small subset of F_1_ iL3s turned out to be homozygous mutants, the pipeline had to be repeated multiple times to obtain statistically significant and sufficiently powered datasets, making it a time-consuming and labor-intensive process. In addition, this approach was inapplicable if both alleles of a gene could not be disrupted in the F_1_ generation, resulting in a lack of homozygous mutant F_1_ iL3s. In some cases, mutant F_1_ iL3s were mosaic for the mutant allele, further complicating phenotypic analysis.

### Innovation

In this protocol, we describe all necessary steps to generate and maintain a stable homozygous knockout line in *S. stercoralis*.^1^^,^^13^ This strategy circumvents the previous need to conduct multiple rounds of the CRISPR pipeline to generate limited numbers of homozygous F_1_ iL3s and enables the generation of large numbers of homozygous mutants of any life stage for phenotypic analyses. This protocol can potentially be used to generate stable homozygous disruptions of any gene in *S. stercoralis* that is not essential for survival or required for the ability to infect a host. In addition, this protocol enables us to maintain stable heterozygous mutant lines in cases where homozygous mutations cause reproductive defects or lethality.

### Institutional permissions

All protocols and procedures involving vertebrate animals must be approved by the Institutional Animal Care and Use Committee (IACUC) and should conform to the standards of the AAALAC and the *Guide for the Care and Use of Laboratory Animals*.

### Generation of CRISPR components

**Timing: 1–2 months**

**Figure 2:**
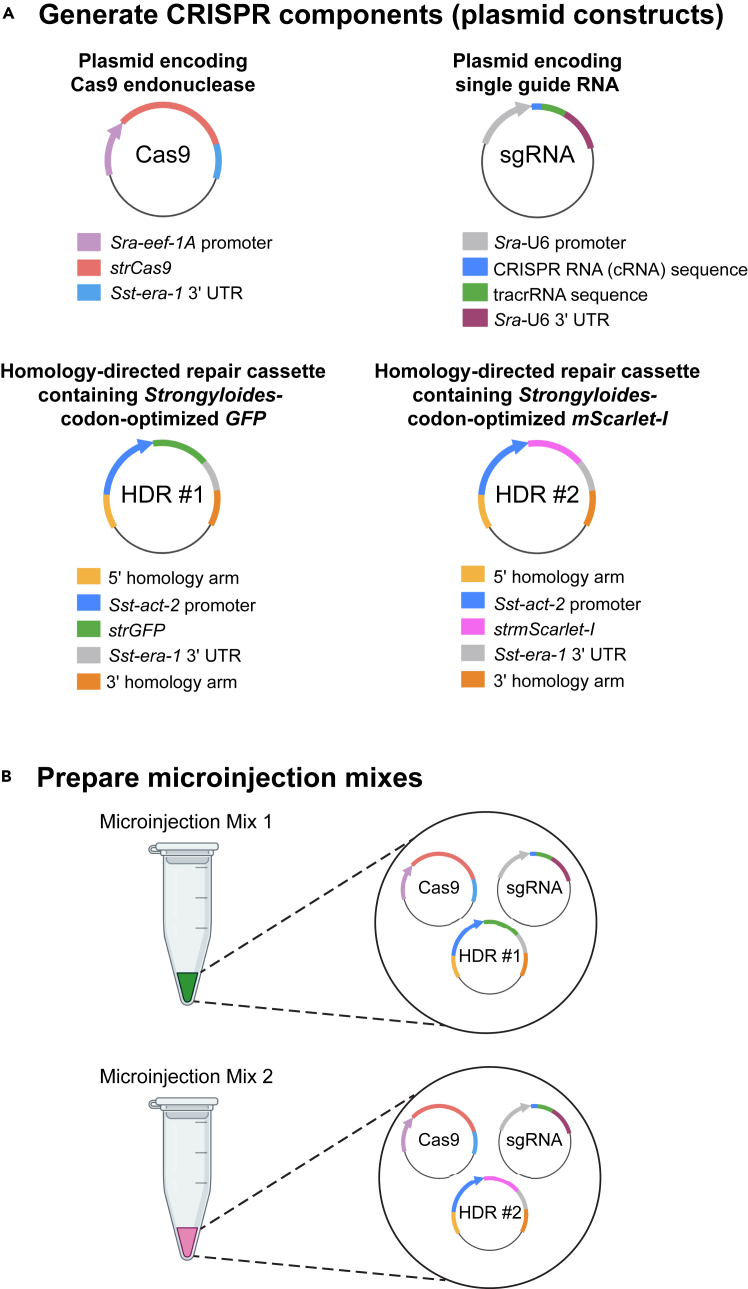
Preparation of CRISPR plasmid constructs (A) The generation of stable knockout lines in *S. stercoralis* requires a plasmid construct for expressing a *Strongyloides*-codon-optimized *Cas9* gene (Cas9), a plasmid construct for expressing a single guide RNA (sgRNA), and two homology-directed repair constructs (HDR #1 and HDR #2) containing genes encoding distinct fluorophores that are codon-optimized for expression in *Strongyloides* (*strGFP* and *strmScarlet-I* are shown, although *strElectra2::*P2A*::strElectra2* can also be used).^1^^,^^13^*Sra-eef-1A* refers to the *S. ratti eef-1a* gene. (B) Two separate microinjection mixes, with each mix containing a single HDR construct encoding a different fluorophore in addition to the Cas9 and the sgRNA expression vectors, are required to generate a stable knockout line.

This section describes how to generate the plasmid constructs required to disrupt a gene locus in *S. stercoralis* using CRISPR/Cas9 technology (Figure 2A).^1^^,^^7^^,^^8^^,^^13^

1. Identify potential Cas9 target sites in the gene of interest that match the consensus sequence 5′-GN(17)GG-3′, which yields efficient gene editing in *C. elegans*,^14^ followed by the Protospacer Adjacent Motif sequence (NGG).

***Note:*** Target sites can be identified using the “Find CRISPR sites” tool in Geneious Prime software.^1^^,^^7^^,^^8^^,^^13^ Target sites near the 5′ end of the gene or in a critical functional domain are preferable. Target sites with higher Doench (2016) activity scores^15^ and the least number of predicted off-target hits are also preferable.

***Note:*** Due to the highly AT-rich genome of *S. stercoralis*, it is often not possible to identify CRISPR sites with the 5′-GN(17)GG-3′ consensus sequence for the gene of interest. In such cases, the criteria may be expanded to include target sites with sequences such as 5′-N(18)GG-3′, 5′-N(19)G-3′ or even 5′-N(20)-3′. Additionally, if a CRISPR site with the 5′-GN(17)GG-3′ consensus sequence is identified but has a low Doench (2016) activity score,^15^ it would be reasonable to select a CRISPR site with a 5′-N(18)GG-3′, 5′-N(19)G-3′ or 5′-N(20)-3′ consensus sequence and a markedly higher Doench (2016) activity score. We consider a Doench (2016) score of 0.6 or greater to be optimal.

2. Generate or obtain a plasmid construct expressing a *Strongyloides*-codon-optimized gene encoding the Cas9 endonuclease (*strCas9*).^1^^,^^6^^,^^7^^,^^8^^,^^13^

***Note:*** We use a plasmid designated pPV540, in which expression of *strCas9* is driven by the *Strongyloides ratti Sra-eef-1A* promoter.^7^^,^^16^

3. Generate a plasmid construct expressing a single guide RNA (sgRNA) for targeting the gene of interest under the control of 500 base pairs (bp) of the *Sra-*U6 promoter and 277 bp of the *Sra-*U6 3′ UTR.

***Note:*** If the gene of interest has two potential Cas9 target sites, then two plasmids – each expressing a sgRNA for one of the cut sites – can be used in tandem.^1^^,^^7^^,^^8^^,^^13^ The sgRNA construct(s) can be commercially synthesized (*e.g.*, by GenScript). Plasmids can be designed using a freely available plasmid editor (*e.g.*, ApE^17^).

4. Generate a plasmid construct expressing an *Sst-act-2p::strGFP* cassette that is flanked by 5′ and 3′ homology arms for homology-directed repair (HDR) of the cut site within the gene of interest.

***Note:*** It is recommended that one of the homology arms maps as close to the cut site as possible (generally within 10 bp of the cut site), while the other homology arm can be up to ∼1 kb from the cut site. When using two sgRNA plasmids to target two distinct Cas9 target sites so as to remove the intervening region, one homology arm should map upstream of the 5′ cut site, while the other homology arm should map downstream of the 3′ cut site.^13^ It is recommended to design homology arms to be ∼500 bp in length, but homology arms can range in size from ∼200–1000 bp. Given the AT-rich nature of the *S. stercoralis* genome, it is often necessary to have the HDR constructs commercially synthesized (*e.g.*, by GenScript). However, if optimal primer binding sites are available, it is possible to PCR-amplify homology arms from genomic DNA.

***Note:*** The closer the homology arms are to the cut site, the higher the efficiency of genomic integration. In addition, homology arms of under 400 bp may reduce the efficiency of genomic integration.

5. Generate a plasmid construct expressing an *Sst-act-2p::strmScarlet-I* cassette that is flanked by 5′ and 3′ homology arms for HDR of the cut site within the gene of interest.

***Note:*** This construct should be identical to the one described above, except with *strmScarlet-I* in place of *strGFP*.

***Note:*** Alternatively, a gene encoding a blue fluorophore, such as *strElectra2*, can be used in one of the HDR constructs in place of either *strGFP* or *strmScarlet-I*.^13^ In this case, it is recommended to use an *Sst-act-2p::strElectra2::*P2A*::strElectra2* construct, since having two copies of *strElectra2* enhances expression and facilitates identification of worms with blue fluorescence^13^ (optional).

***Note:****Sst-act-2p* refers to the promoter sequence of the *S. stercoralis act-2* gene, which drives gene expression in body-wall muscle. CRISPR integration of the repair cassette within the genomic locus results in worms expressing the fluorophores throughout all or nearly all of the body-wall muscle, allowing visual identification of candidate knockouts.^7^*strGFP* and *strmScarlet-I* refers to the sequences of the *Strongyloides*-codon-optimized *GFP* and *mScarlet-I* genes, respectively.

**CRITICAL:** It is important to codon-optimize the *Cas9* endonuclease gene as well as the fluorophore genes in the HDR constructs based on codon usage patterns in *Strongyloides*.^18^ This greatly enhances gene expression levels and facilitates screening for worms with integrated repair cassettes, which generally show bright expression of the fluorophores throughout the body-wall muscle.

***Note:*** The generation of stable homozygous knockout lines in *S. stercoralis* requires the generation of two HDR constructs, each expressing a gene encoding a different fluorophore. This enables the visual identification of homozygous knockout worms in the F_3_ generation because these worms co-express two distinctly colored fluorophores. A detailed explanation of this principle is described in later sections.

### Preparation of microinjection mixes

**Timing: 15–30 min**

This section describes how to prepare microinjection mixes for intragonadal microinjection of free-living females. The generation of stable homozygous knockout lines in *S. stercoralis* requires preparation of two different microinjection mixes. While both mixes contain plasmid constructs for expression of the Cas9 endonuclease and the sgRNA, one mix contains an HDR plasmid construct for driving expression of *strGFP* (HDR #1) and the other mix contains an HDR plasmid construct for driving expression of *strmScarlet-I* (HDR #2) (Figure 2B).

6. Determine the concentration of all four plasmid stocks using a Nanodrop or similar microvolume spectrophotometer.
7. Prepare Microinjection Mix 1:
  a. Dilute the Cas9, sgRNA and HDR #1 plasmids in BU saline^19^ or ddH_2_O to a total volume of 10–20 μL to achieve the desired concentrations in the mix (Table 1).
  b. Spin the mix through a filter column (0.22 μm pore size) at 16200 × *g* for 1–2 min.
8. Prepare Microinjection Mix 2:
  a. Dilute the Cas9, sgRNA and HDR #2 plasmids in BU or ddH_2_O to a total volume of 10–20 μL to achieve the desired concentrations in the mix (Table 2).
  b. Spin the mix through a filter column (0.22 μm pore size) at 16200 × *g* for 1–2 min.

***Note:*** It is recommended to determine the total DNA concentration in the microinjection mixes after spinning them through the columns using a Nanodrop or similar microvolume spectrophotometer. Microinjection mixes that are within 10–20 ng/μL of the expected total DNA concentration are acceptable for use.

9. Use the microinjection mixes on the same day or store them at −20°C.

***Note:*** If using two sgRNA constructs, each targeting distinct CRISPR sites within the same gene, add the constructs to the microinjection mix at a final concentration of 40 ng/μL each.

**Table 1:** Components of Microinjection Mix 1 for disrupting the *Sst-gcy-9* locus

Plasmid name	Description	Concentration in mix
pPV540	Construct for expression of *Strongyloides*-codon-optimized *Cas9*	50 ng/μL
pSSG05	Single guide RNA construct for CRISPR-mediated disruption of *Sst-gcy-9*	80 ng/μL
pMLC175	Homology-directed repair construct for *Sst-gcy-9* containing a *Strongyloides*-codon-optimized *GFP* gene	80 ng/μL

**Table 2:** Components of Microinjection Mix 2 for disrupting the *Sst-gcy-9* locus

Plasmid name	Description	Concentration in mix
pPV540	Construct for expression of *Strongyloides*-codon-optimized *Cas9*	50 ng/μL
pSSG05	Single guide RNA construct for CRISPR-mediated disruption of *Sst-gcy-9*	80 ng/μL
pMLC176	Homology-directed repair construct for *Sst-gcy-9* containing a *Strongyloides*-codon-optimized *mScarlet-I* gene	80 ng/μL

### Culturing *S. stercoralis* to obtain free-living adults for microinjection

**Timing: 1–2 days**

**Figure 3:**
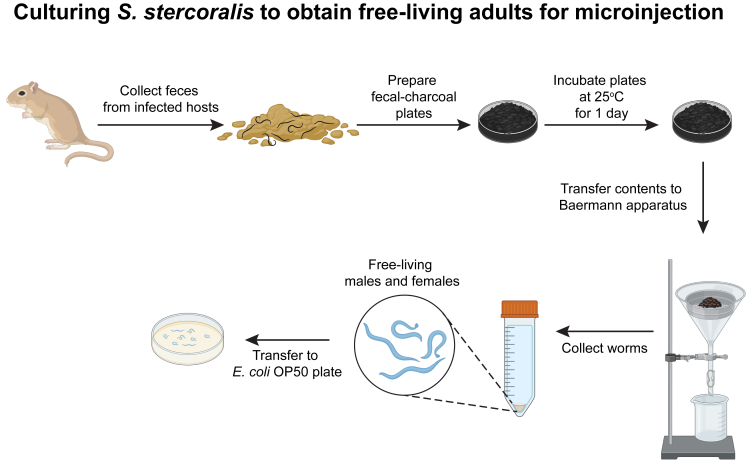
Culturing and isolation of free-living adults for microinjection Workflow for obtaining *S. stercoralis* free-living males and females for microinjecting CRISPR components.

This section describes how to obtain free-living *S. stercoralis* females and males (Figure 3). The CRISPR components will be introduced into free-living females by intragonadal microinjection,^20^ and the females will then be mated with uninjected free-living males. It is also possible to introduce exogenous DNA into free-living males,^21^ although this is not necessary to obtain homozygous mutants and is not routinely done.

10. Prepare fecal-charcoal cultures:
  a. Collect feces from infected hosts (Mongolian gerbils) in an ABSL-2 vivarium the day before microinjection.
    i. Two days before microinjection, house gerbils in a cage with a wire cage bottom placed atop a base layer of wet cardboard.
      ***Note:*** It is important to moisten the entire surface of the cardboard to prevent desiccation of worms within the fecal pellets.
    ii. After ∼12–14 h, transfer gerbils to a fresh cage with bedding. Set aside.
    iii. Remove the wire cage bottom from the old cage, leaving the feces-covered wet cardboard in place.
    iv. Using a plastic spoon, collect fecal pellets from the wet cardboard and place into a plastic bag.
  b. Transport the plastic bag containing feces from the ABSL-2 vivarium to the laboratory.
  c. In the laboratory, transfer feces to a disposable plastic cup.
  d. Moisten feces with water.
  e. Add a volume of autoclaved charcoal roughly equivalent to 3× the volume of feces and mix thoroughly using a tongue depressor.
    ***Note:*** The feces-to-charcoal ratio can be adjusted depending on the expected worm output and the amount of feces collected. In general, feces from 8 gerbils can be used to make three 10-cm plates with a 1:3 feces-to-charcoal ratio. However, with a strong infection, less feces can be used per plate.
  f. Continue to add water to the fecal-charcoal mix until the charcoal surface looks shiny.
    ***Note:*** This will prevent the feces from drying out, which can result in desiccation of the worms.
  g. Line the bottom of a 10-cm Petri dish with a circular piece of Whatman filter paper of equivalent diameter and add enough water to dampen the Whatman filter paper.
  h. Transfer the fecal-charcoal mix to the Petri dish, cover with a lid, and incubate in a secondary container lined with a moist paper towel at 25°C for 1 day to obtain free-living adults for microinjection.
  i. Alternatively, incubate the fecal-charcoal plate at 20°C for 2 days to obtain free-living adults for microinjection (optional).

### Preparation of microinjection pads

**Timing: 1 day**

This section describes how to prepare microinjection pads for immobilizing the worms during intragonadal microinjection.

11. Prepare agarose pads for microinjection:
  a. Prepare a 5% solution of agarose in ddH_2_O in a glass test tube.
  b. Heat the 5% agarose solution over a Bunsen burner until boiling and all agarose is dissolved. While pouring agarose onto coverslips, leave the tube with agarose solution in a heat block set to 95°C.
  c. Using a glass Pasteur pipet, transfer ∼180 μL of the agarose solution onto a 48 × 60 mm coverslip.
  d. Immediately drop a second coverslip on top to make a thin agarose pad.
  e. Remove the second coverslip after 5–10 s by sliding the two coverslips apart.
  f. Dry the agarose pads for 16–24 h at 22°C–23°C.

***Note:*** Prepare the agarose pads at least 1 day before microinjection. The pads can also be made well in advance and stored at 22°C–23°C for up to 2 months. It is recommended to not use the same agarose pad for more than one day of microinjection.

### Preparation of fecal-charcoal plates made with uninfected feces

**Timing: 15 min**

This section describes how to make fecal-charcoal plates using feces from uninfected gerbils. These plates should be freshly made on the day of microinjection. After microinjection, the microinjected free-living *S. stercoralis* females and non-microinjected free-living *S. stercoralis* males will be transferred to these plates. In cases where it is necessary to cross heterozygous free-living males and females to generate homozygous knockouts (see below), the mated free-living males and females will also be transferred onto fecal-charcoal plates made with uninfected feces.

12. Prepare fecal-charcoal cultures:
  a. Collect feces from uninfected hosts (Mongolian gerbils) in an ABSL-2 vivarium on the day of microinjection, as described above (step 10).
  b. Mix feces with water and charcoal, as described above (step 10), except that the volume of autoclaved charcoal should be roughly equivalent to the volume of feces.
  c. Prepare a fecal-charcoal plate, as described above (step 10), using a 6-cm Petri dish.

## Key resources table

**Table:** 

REAGENT or RESOURCE	SOURCE	IDENTIFIER
**Bacterial and virus strains**		

*Escherichia coli* OP50	Caenorhabditis Genetics Center	OP50

**Chemicals, peptides, and recombinant proteins**		

Agar	Fisher Scientific	BP1423-2
Agarose, molecular biology grade	Research Products International (RPI)	A20090500.0
Amphotericin B	Thermo Fisher Scientific (Gibco)	15290–018
Bacto Peptone	BD	211677–500
Boric acid	Sigma-Aldrich	B6768-1KG
CaCl_2_	Fisher Scientific	BP510-500
Charcoal (Bone Char)	Ebonex	EBO.58BC
Cholesterol	Thermo Scientific	A11470-18
dNTP mix	NEB	N0447S
Dulbecco’s modified Eagle’s medium (DMEM)	Thermo Fisher Scientific (Gibco)	11995–065
Ethanol, 190 proof	Decon Laboratories	2801
Ethylenediaminetetraacetic acid, disodium salt dihydrate (EDTA)	Fisher Scientific	S311-500
Gelatin	Fisher Scientific	G8-500
GelGreen nucleic acid gel stain	Biotium	41005
Halocarbon oil 700	Sigma-Aldrich	H8898-50ML
IGEPAL CA-630	US Biologicals	9036-19-5
KCl	Fisher Scientific	BP366-500
KH_2_PO_4_	Fisher	P288-500
KPO_4_	Fisher	P285-500
2-mercaptoethanol	MP Biomedicals	0219483425
MgCl_2_	Fisher Scientific	BP214-500
MgSO_4_	Fisher Scientific	M63-500
NaCl	VWR (J.T. Baker)	JT3624-15
NaOH	Fisher Scientific	S318-500
Na_2_HPO_4_	Fisher	S374-500
Nematode growth medium (NGM)	Research Products International (RPI)	N81800–1000.0
Nicotine	Sigma-Aldrich	N3876-5ML
Penicillin/Streptomycin	Thermo Fisher Scientific (Gibco)	15140–122
Phosphate-buffered saline (PBS)	Thermo Fisher Scientific (Gibco)	10010–023
Platinum Taq DNA polymerase PCR kit	Thermo Fisher Scientific	10966034
Proteinase K	MP Biomedicals	0219350480
Tetracycline hydrochloride solution	Zymo Research	A1004-5
Tris pH 8.0	Thermo Fisher Scientific (Invitrogen)	AM9855G
Tris Base	Fisher Scientific	BP152-500
Tryptone	Fisher Scientific	BP1421-500
Tween 20	Fisher Scientific	BP337-100
Yeast extract	Fisher Scientific	BP1422-500

**Experimental models: Organisms/strains**		

*Strongyloides stercoralis* Strain: UPD Sex: males and females of free-living adults, female infective larvae Age: isolated from 1- to 2-day-old plates (for free-living adults) or 7- to 10-day-old plates (for infective larvae)	Maintained in lab	UPD (wild type)
*Strongyloides stercoralis* Strain: EAH484 Sex: female infective larvae Age: isolated from 7- to 10-day-old plates (for infective larvae)	Banerjee et al.^1^	*Sst-gcy-9*^−/−^ (red/green dual color)
*Meriones unguiculatus*: Mongolian gerbils Strain: 243 Sex: male Age: ∼1.5–3 months	Charles River Laboratories	Mongolian gerbil strain 243

**Oligonucleotides**		

AATCTTAAATCAAAAGGTGG	Banerjee et al.^1^	*Sst-gcy-9* CRISPR target sequence
**SG78 (“*Sst-act-2* exon 1 F1”)** 5′-GTA TTC CCT TCT ATT GTT GGA AGA CC-3′	Banerjee et al.^1^; Gang et al.^7^	Primer for amplifying exon 1 of the *Sst-act-2* gene
**SG80 (“*Sst-act-2* exon 1 R1”)** 5′-CCT TCA TAG ATT GGT ACA GTG TGA G-3′	Banerjee et al.^1^; Gang et al.^7^	Primer for amplifying exon 1 of the *Sst-act-2* gene
**SG142 (“*Sst-gcy-9.1* F1”)** 5′-GGA TAC TTT GTC AAC GTG GTT CAT T-3′	Banerjee et al.^1^	Primer for amplifying the *Sst-gcy-9* wild-type locus near the CRISPR target site *-and-* Primer that flanks the 5′ homology arm for testing for genomic integration of the homology-directed repair cassette
**SG143 (“*Sst-gcy-9.1* R1”)** 5′-GAC TTC CAC CAC CTT TTG ATT TAA GA-3′	Banerjee et al.^1^	Primer for amplifying the *Sst-gcy-9* wild-type locus near the CRISPR target site
**MLC95 (“*Ss* mutscreen R2”)** 5′-CGA GGT ACC TCT TTT CCA CAC TT-3′	Banerjee et al.^1^	Primer that flanks the 5′ homology arm for testing for genomic integration of the homology-directed repair cassette

**Recombinant DNA**		

**pPV540** *Sra-eef-1Ap::strCas9::Sst-era-1* 3′ UTR	Shao et al..^16^; Gang et al.^7^	Construct for expression of *Strongyloides-*codon-optimized *Cas9*
**pSSG05** *Sra-U6* 5′ UTR*::Sst-gcy-9-sgRNA::Sra-U6* 3′ UTR	Banerjee et al.^1^	Single guide RNA construct for CRISPR-mediated disruption of *Sst-gcy-9*
**pMLC175 (HDR #1)** 5′ HA::*Sst-act-2p::strGFP*::*Sst-era-1* 3′ UTR::3′ HA	Banerjee et al.^1^	Homology-directed repair construct for *Sst-gcy-9* containing a *Strongyloides*-codon-optimized *GFP* gene
**pMLC176 (HDR #2)** 5′ HA::*Sst-act-2p::strmScarlet-I*::*Sst-era-1* 3′ UTR::3′ HA	Banerjee et al.^1^	Homology-directed repair construct for *Sst-gcy-9* containing a *Strongyloides*-codon-optimized *mScarlet-I* gene

**Software and algorithms**		

Geneious Prime	Dotmatics	https://www.geneious.com/
Zeiss Zen 3.3 (blue edition)	Carl Zeiss	
Fiji	Schindelin et al.^22^	https://fiji.sc/
ApE: A plasmid Editor	Davis and Jorgensen^17^	https://jorgensen.biology.utah.edu/wayned/ape/

**Other**		

Aluminum foil	Fisher Scientific	01-213-105
Baermann apparatus	Castelletto and Hallem^20^, Lok et al.^23^	N/A
Bunsen burner	VWR	89038–526
Cardboard (8 × 16.5 in)	Newco Specialty (Shepherd Specialty Papers Techboard)	999589N
Centrifuge	Eppendorf	Centrifuge 5702 R
Centrifuge	Thermo Fisher Scientific (Sorvall)	Legend Micro 21
Centrifuge tube filter (0.22 μm)	Corning	Costar® Spin-X 8160
Conical tube (50 mL)	VWR	89-39-656
Conical tube (15 mL)	Corning	430791
Cover glass (48 × 60 mm, No. 1 thickness)	Brain Research Laboratories	4860-1D
Feeding tube, plastic (for oral gavage)	Instech Laboratories	FTP1850
Gel electrophoresis apparatus	Thermo Fisher Scientific	OWL D3-14
Gel electrophoresis power supply	Fisher Scientific	FB300
Gel imaging system	Bio-Rad	ChemiDoc XRS+
Glass bottles (for media storage)	DWK Life Sciences (Wheaton)	219815
Glass capillaries	World Precision Instruments	1B120F-4
Glass test tubes	Fisher Scientific	14-961-27
Heat block (dry)	VWR	75838–282
Hot plate (stirring)	Thermo Fisher Scientific(Cimarec+)	SP88857100
Incubator	VWR	89510–746
Incubator, CO_2_	Eppendorf (New Brunswick)	Galaxy 48 R CO48HK600912
Microcentrifuge tube (1.5 mL)	VWR	89000–028
Microcentrifuge tube (1.5 mL, screw cap)	VWR	16466–062
Microinjector (coarse manipulator)	Narishige	MMN-1
Micromanipulator (micromanipulator)	Narishige	MMO-4
Microvolume spectrophotometer	Thermo Fisher Scientific	NanoDrop One/One^c^
Microscope, dissection	Leica	Ivesta 3
Microscope, fluorescence	Zeiss	AxioObserver 7
Microscope, fluorescence dissection	Leica	M165 FC
Microscope, microinjection	Zeiss	AxioObserver A1
Multi-well plate (96 wells)	Thermo Fisher Scientific (Nunc)	268200
Needle puller	Sutter Instrument Company	P-30
Paintbrush (nail liner brush)	Symphony	053742756612
Paper towels	Kimberly-Clark (Scott)	06041
Pasteur pipets	Fisher Scientific	13-678-20A
PCR tube (0.2 flat cap)	Fisher Scientific (Axygen)	14-222-262
Peristaltic pump	Wheaton	Unispense
Petri dish (100 × 15 mm), vented (for LB plates)	VWR	25384–342
Petri dish (60 × 15 mm), vented (for fecal-charcoal and NGM plates)	Tritech Research	T3315
Petri dish (100 × 20 mm), glass bottom (for secondary containment)	VWR	75845–524
Petri dish (100 × 20 mm), glass top (for secondary containment)	VWR	75845–514
Petri dish (100 × 20 mm), vented (for fecal-charcoal plates)	Greiner Bio-One	664102
Pipettor (P20), autoclavable	VWR	83009–762
Pipettor (P200), autoclavable	VWR	83009–770
Pipettor (P1000), autoclavable	VWR	83009–776
Pipet tips, P20, low binding	Genesee Scientific, Olympus Plastics	23–404
Pipet tips, P200, low binding	Genesee Scientific, Olympus Plastics	23–412
Pipet tips, P1000, low binding	Genesee Scientific, Olympus Plastics	23–430
Pipet controller	Integra Biosciences	PIPETBOY acu 2
Pipets, serological	Thermo Fisher Scientific (Nunc)	170356N
Plastic box, 1.75 L (secondary container for storing fecal-charcoal plates)	Really Useful Products	Really Useful Box, 1.75 L
Plastic spoons (for fecal collection)	Stalk Market	812855012165
Plastic bags (for fecal collection)	Fisher Scientific	14-955-189
Plastic cups (for fecal-charcoal preparation)	Office Depot (Highmark)	697019
Platinum Wire, 36 Gauge (for worm pick)	Thomas Scientific	1233S72
Syringe, disposable (1 mL, Luer-Lok tip)	BD (Becton Dickinson)	309628
Thermocycler	Eppendorf	Mastercycler Nexus gradient thermal cycler 6336000023
Tongue depressors (for fecal-charcoal preparation)	Puritan Medical Products	704
Filter paper (90 mm)	Cytiva (Whatman)	1001090
Filter paper (55 mm)	Cytiva (Whatman)	1001055
Watch glass (small)	Carolina Biological Supply	742300
X-ACTO Knife (worm pick holder)	Amazon	X3001

## Materials and equipment

**Table:** Worm Buffered Saline (BU) solution (1×)

Reagent	Final concentration	Amount
Na_2_HPO_4_	50 mM	7.10 g
KH_2_PO_4_	22 mM	2.99 g
NaCl	70 mM	4.09 g
ddH_2_O	N/A	to 1 L
**Total**	**N/A**	**1 L**

• Make 25–50 mL aliquots.
• Autoclave.

Storage condition: Store at 22°C–23°C.

**Table:** 2% Nematode Growth Medium (NGM) plates

Reagent	Final concentration	Amount
NaCl	51.3 mM	3 g
Agar	2% (w/v)	20 g
Peptone	0.25% (w/v)	2.5 g
ddH_2_O	N/A	975 mL
Autoclave for 50 min. Cool to 55°C		
1 M CaCl_2_	1 mM	1 mL
5 mg/mL cholesterol in ethanol	5 μg/mL	1 mL
1 M MgSO_4_	1 mM	1 mL
1 M KPO_4_	25 mM	25 mL
**Total**	**N/A**	**1 L**

• Pour plates using a peristaltic pump.
• Dispense 10 mL into each 6-cm plate.
• Let plates dry at 22°C–23°C for at least a day before use.

Storage conditions for all reagents: Store reagents at room temperature.

Storage conditions for plates: Store plates lid-side-down at 22°C–23°C for up to 2 weeks or at 4°C for 3–4 months.

**Table:** 2% Nematode Growth Medium (NGM) plates (from commercial media)

Reagent	Final concentration	Amount
NGM	2.3% (w/v)	23 g
agar	0.25% (w/v)	2.5 g
ddH_2_O	N/A	975 mL
Autoclave for 50 min. Cool to 55°C.		
1 M CaCl_2_	1 mM	1 mL
1 M MgSO_4_	1 mM	1 mL
1 M KPO_4_	25 mM	25 mL
**Total**	**N/A**	**1 L**

• Pour plates using a peristaltic pump.
• Dispense 10 mL into each 6-cm plate.
• Let plates dry at 22°C–23°C for at least a day before use.

Storage conditions for all reagents: Store reagents at room temperature.

Storage conditions for plates: Store plates lid-side-down at 22°C–23°C for up to 2 weeks or at 4°C for 3–4 months.

**Table:** LB plates

Reagent	Final concentration	Amount
NaCl	170 mM	5 g
tryptone	1.0% (w/v)	10 g
yeast extract	0.5% (w/v)	5 g
agar	1.5% (w/v)	15 g
1 N NaOH	1 mM	1 mL
ddH_2_O	N/A	to 1 L
**Total**	**N/A**	**1 L**

• Autoclave.
• Let cool for a few minutes on a hot plate with stirring and heat set to 60°C.
• Pour plates using a peristaltic pump. Dispense 20 mL into each 10-cm plate.
• Let plates cool at 22°C–23°C overnight, then store at 4°C.

Storage conditions for all reagents: Store reagents at 22°C–23°C.

Storage conditions for plates: Store plates lid-side-down at 4°C for 3–4 months.

**Table:** LB broth

Reagent	Final concentration	Amount
NaCl	0.17 M	5 g
tryptone	1.0% (w/v)	10 g
yeast extract	0.5% (w/v)	5 g
1 N NaOH	1 mM	1 mL
ddH_2_O	N/A	to 1 L
**Total**	**N/A**	**1 L**

• Aliquot into 75–100 mL aliquots.
• Autoclave.

Storage condition: Store at 22°C–23°C.

### *Escherichia coli* OP50 stock solution

• Pick a single colony from a lawn of *E. coli* OP50 bacteria on an LB plate.
• Inoculate 75–100 mL LB media using a sterilized inoculation loop.
• Incubate the media for 16–18 h (without shaking) at 37°C.
• Store at 4°C for 1 month.

### *E. coli* OP50 plates

• Add 150–200 μL of *E. coli* OP50 stock solution onto each NGM plate.
• Allow plates to dry at 22°C–23°C for 2–3 days.
• Store seeded NGM plates lid-side-down at 22°C–23°C for up to 2 weeks.

### Tetracycline hydrochloride (antibiotic), 2000× stock solution

• Make 50 μL aliquots of a commercially pre-made stock solution of tetracycline hydrochloride (10 mg/mL).
• Store at −20°C.

**CRITICAL:** Since tetracycline hydrochloride is light sensitive, it is important to store the aliquots in opaque microcentrifuge tubes or tubes shielded in aluminum foil.

### Penicillin/streptomycin (antibiotic), 100× stock solution

• Make 500 μL aliquots of a commercially pre-made stock solution of penicillin/streptomycin (10,000 U/mL penicillin, 10,000 μg/mL streptomycin) in microcentrifuge tubes.
• Store at −20°C for up to 12 months from date of manufacture.

### Amphotericin B (antifungal), 100× stock solution

• Make 500 μL aliquots of a commercially pre-made stock solution of amphotericin B (250 μg/mL) in microcentrifuge tubes.
• Store at −20°C for up to 12 months from date of manufacture.

**Table:** 1× Phosphate-buffered saline (PBS) solution

Reagent	Final concentration	Amount
NaCl	137 mM	8 g
KCl	2.7 mM	0.2 g
Na_2_HPO_4_	10 mM	1.15 g
KH_2_PO_4_	1.8 mM	0.2 g
Adjust pH to 7.4		
ddH_2_O	N/A	to 1 L
**Total**	**N/A**	**1 L**

• Make 25–50 mL aliquots.
• Autoclave.

Storage condition: Store at 22°C–23°C for up to 1 year.

Alternatively, commercially available 1× PBS solution can be used (optional).

**Table:** Worm lysis buffer (without proteinase K and 2-mercaptoethanol)

Reagent	Final concentration	Amount
KCl	50 mM	25 mL
Tris pH 8.0	10 mM	5 mL
MgCl_2_	2.5 mM	1.25 mL
IGEPAL CA-630	0.45%	2.25 mL
Tween 20	0.45%	2.25 mL
gelatin	0.01%	0.05 g
ddH_2_O	N/A	464.25 mL
**Total**	**N/A**	**500 mL**

• Heat at ∼100°C for ∼15 min until ingredients dissolve.
• Filter-sterilize, aliquot, and store at 4°C.
• Before use, add proteinase K and 2-mercaptoethanol according to the table below.

Storage condition: Store lysis buffer at 4°C.

**Table:** Worm lysis buffer (with proteinase K and 2-mercaptoethanol)

Worm lysis buffer	N/A	167 μL
proteinase K (10 mg/mL)	0.12 mg/mL	2 μL
2-mercaptoethanol	1.74%	3 μL

Storage condition: Store proteinase K at −20°C.

Storage condition: Store 2-mercaptoethanol at 22°C–23°C.

**CRITICAL:** 2-mercaptoethanol is corrosive, flammable and toxic if swallowed, inhaled or absorbed through skin. Use in a fume hood and wear gloves.

### Proteinase K solution

• Prepare a 10 mg/mL stock solution of proteinase K in ddH_2_O.
• Prepare aliquots of 50 μL.
• Store at −20°C.

### Nicotine solution

• Prepare a 10% solution of nicotine in ddH_2_O.
• Dilute to a final concentration of 1% or 2% in BU saline.
• Store at 22°C–23°C.

**Table:** 1× TBE buffer

Reagent	Final concentration	Amount
Tris base	0.13 M	10.8 g
boric acid	45 mM	5.5 g
EDTA	2.5 mM	0.93 g
ddH_2_O	N/A	to 1 L
**Total**	**N/A**	**1 L**

• Storage condition: Store at 22°C–23°C.

## Step-by-step method details

### Microinjection of CRISPR components

**Timing: 2 days**

**Figure 4:**
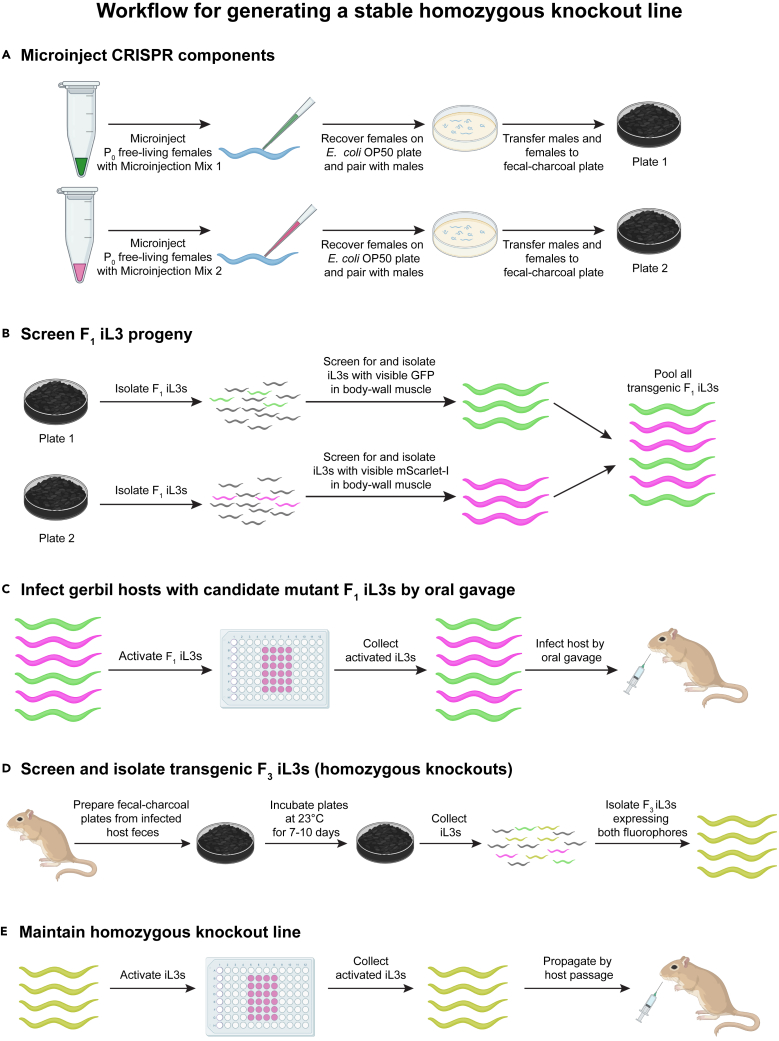
Workflow for generating and maintaining a stable homozygous knockout line of *S. stercoralis* (A) Microinjection of CRISPR constructs into P_0_ free-living females. (B) Screening and isolation of F_1_ iL3 progeny based on whole-body expression of a body-wall muscle marker. Note that in the F_1_ generation, candidate knockouts are visually identified as iL3s that are fluorescent across their entire body-wall muscle, which is the site of expression of the *Sst*-*act-2* promoter. (C) Passage of F_1_ iL3s through a host. (D) Screening and isolation of F_3_ homozygous knockout iL3s based on coexpression of red and green body-wall muscle markers. (E) Passage of homozygous knockout iL3s through a host and maintenance of a stable knockout line. In B–E, pink worms represent worms expressing mScarlet-I; green worms represent worms expressing GFP; yellow worms represent worms expressing both mScarlet-I and GFP; and gray worms represent non-transgenic worms.

This section describes how to microinject CRISPR plasmid constructs into the germline of *S. stercoralis* free-living females (Figures 3 and 4A). For a more detailed step-by-step description of the process, please refer to Castelletto and Hallem, 2021.^20^

1. Prepare worms for microinjection (1–3 h):
  a. To collect free-living adults for microinjection, set up a Baermann apparatus^20^^,^^23^ with a fecal-charcoal plate that has been incubating at 25°C for 1 day or at 20°C for 2 days.
  b. After 1–2 h, collect 30–40 mL of liquid containing free-living adults from the Baermann apparatus into a 50 mL conical tube.
  c. To concentrate worms, transfer ∼10 mL of liquid from the 50 mL conical tube into a 15 mL conical tube.
  d. Centrifuge the 15 mL conical tube at ∼3000 × *g* for 1 min to pellet worms.
  e. Alternatively, allow the worms in the 50 mL tube to settle by gravity for 10–15 min (optional).
  f. Aspirate the supernatant, leaving behind the worm pellet.
  g. If needed, repeat steps c-d to obtain a decent-sized worm pellet (∼50–100 μL volume).
  h. Transfer worms in as little liquid as possible to an *E. coli* OP50 plate.^24^
  i. Wait for the liquid to dry out and the worms to settle on the bacterial lawn.
  j. Using a worm pick, transfer ∼50–100 worms to an unseeded NGM plate^24^ to remove excess bacteria and enable selection of single worms for microinjection.

2. Microinject free-living females (4–6 h):
  a. Prepare microinjection needles and load each needle with 0.7–1 μL of microinjection mix.
    ***Note:*** Make 2–3 microinjection needles at a time for each microinjection mix since the needles may get clogged during injection and will need to be replaced.
  b. After attaching the needle to the microinjector connected to the microinjection microscope, break the tip of the needle.
    i. Place a glass shard on the dry agarose surface of a microinjection pad.
    ii. Cover with a droplet of halocarbon oil, such that the glass shard is fully submerged.
    iii. Observe the needle tip under the microscope and gently drive the needle tip against the glass shard, allowing fluid to flow from the needle tip.
  c. Using a worm pick or a paintbrush, transfer a free-living female from the unseeded NGM plate onto a drop of halocarbon oil on the agarose surface of the microinjection pad.
    ***Note:*** Young free-living adult females with 1–4 eggs in their gonad usually work well for microinjections, although individual preference regarding the age of the adults may vary.
    ***Note:*** To expedite the process, more than one free-living female can be transferred to the agarose pad simultaneously and microinjected in succession. Free-living females are rarely viable once desiccated, so all worms injected in succession must be recovered before they dry out (*i.e.,* within 5 min of placement on the agarose pad).
  d. Insert the microinjection needle into a gonad of a free-living female and fill the gonad arm with the microinjection mix. Repeat for the other gonad, if accessible.
  e. Microinject other worms on the agarose pad, if any (optional).
  f. To recover worms from the pad, add a ∼2 μL droplet of BU saline on top of the worms to release them from the pad.
  g. Using a paintbrush, transfer the worms from the BU droplet to an *E. coli* OP50 plate.
    ***Note:*** It is recommended that worms be placed on the NGM surface, directly adjacent to the border of the bacterial lawn.
  h. Microinject 75–150 worms with Microinjection Mix 1.
    **CRITICAL:** It is important to estimate the number of free-living females to inject by performing pilot microinjection runs before attempting to make a stable line. Pilot microinjection runs for Microinjection Mix 1 and Microinjection Mix 2 can be performed using a small number of P_0_ free-living females (approximately 20 females per mix). This will allow estimation of the rate of F_1_ transgenesis and the rate of integration of the repair cassette for each mix. To visually estimate the number of F_1_ iL3 progeny with integration of the HDR cassette, tally the number of F_1_ iL3 progeny expressing the fluorophore in their body-wall muscle along at least 80% of their body length. This will also allow calculation of the number of free-living females required to inject during the stable line microinjection run (*n.b.,* the goal is at least ∼100 iL3s with integrated repair cassettes of each fluorophore). It may also be helpful in the pilot runs to genotype (see below) these iL3s to determine what fraction have integrated repair cassettes.
  i. Recover all microinjected females onto a single fresh *E. coli* OP50 plate.
  j. Transfer free-living males to the same recovery plate with the microinjected females.
    ***Note:*** Place approximately 1 male per 2 injected females on the recovery plate.
  k. Allow the worms to recover and mate on this plate for at least 30 min.
  l. Flood the recovery plate with water or BU saline and then use a P20 micropipette to serially transfer the injected females as well as the males (in 5–20 μL liquid per transfer) to a 10-cm fecal-charcoal plate. Place the plate under a dissection microscope during the transfer in order to see the worms.
    ***Note:*** The 10-cm fecal-charcoal plate is freshly made on the day of microinjection, from uninfected gerbil feces, as described above.
    ***Note:*** It is recommended to use the same pipet tip for transferring all the adults from a single *E. coli* OP50 plate onto the fecal-charcoal plate. Once all adults have been transferred, determine whether any worms are stuck to the pipet tip by briefly pipetting up and down in a watch glass filled with water or BU saline under the microscope. Any worms that were stuck to the pipet tip will be flushed out and can be transferred onto the fecal-charcoal plate.
  m. Place the fecal-charcoal plate in a secondary container lined with a damp paper towel and incubate at 23°C for 4–8 days.
  n. The day after microinjecting using Microinjection Mix 1, microinject using Microinjection Mix 2, as described above.
  o. Recover these injected free-living females and non-injected free-living males to a separate fecal-charcoal plate, made fresh that day from uninfected gerbil feces, as described above.

### Screening of F_1_ iL3 progeny

**Timing: 7–9 h**

This section describes the steps required to screen and isolate F_1_ transgenic progeny of microinjected free-living females of *S. stercoralis* (Figure 4B).

3. Collect F_1_ iL3 progeny from microinjected females.
  a. Set up two Baermann apparatuses, one for each batch of microinjected worms.
  b. After 2.5–3 h, collect 40–50 mL of liquid containing F_1_ iL3 progeny from each Baermann apparatus into two separate 50 mL conical tubes.
    ***Note:*** To expedite the screening process, it is possible to collect liquid from the Baermann apparatus after 1 h and then start screening the worms that have already collected in the tube. The Baermann apparatus can then be left up for an additional 1.5–2 h to collect additional worms if needed.
  c. Concentrate worms in 1.5 mL of liquid in separate 15 mL tubes, as described above (step 1).
  d. Transfer the worm suspension from the 15 mL tube to a microcentrifuge tube and then further concentrate worms in 100–300 μL of liquid.
    ***Note:*** For concentrating worms in microcentrifuge tubes, spin at ∼3000 × *g* for 1–2 min.

4. Screen and isolate F_1_ iL3s with potential gene disruptions.
  a. Depending on density, transfer subsets of iL3s in 2–10 μL droplets onto *E. coli* OP50 plates.
    ***Note:*** It is recommended to use NGM plates with thick lawns of OP50 bacteria. *S. stercoralis* iL3s are highly active on plates, and the thick bacterial lawn helps to slow them down.
    ***Note:*** If resources permit, it is recommended to have at least two individuals screening F_1_ iL3s in parallel to make the screening process more efficient.
    **CRITICAL:** It is important to optimize the density of iL3s on the screening plates since too high a density may lead to accidental selection of worms that are not potential mutants. Use a fresh *E. coli* OP50 plate for each 2–10 μL droplet of worms being screened. In addition, it is recommended not to paralyze iL3s with nicotine for screening. In our experience, worms treated with nicotine do not activate in the *in vitro* activation assay.
    ***Note:*** From the free-living females injected with either Microinjection Mix 1 or Microinjection Mix 2, a subset of the transgenic iL3 progeny will express the fluorophore (GFP or mScarlet-I) in body-wall muscle almost throughout their entire body length. These transgenic iL3 progeny likely have HDR cassettes that have integrated into either one (heterozygous) or both (homozygous) alleles of a gene, allowing for fluorophore expression in the majority of body-wall muscle cells. A second subset of iL3s will show patchy expression of the fluorophore along their body length. These transgenic iL3 progeny generally express the repair cassettes as non-integrated (extrachromosomal) transgenes and are less likely to contain gene disruptions. After the F_1_ generation, extrachromosomal arrays are silenced in *S. stercoralis*^25^; thus, all fluorophore expression in subsequent generations comes from integrated repair cassettes.
  b. Place the NGM plate under a fluorescence dissection microscope to detect transgenic iL3s.
    ***Note:*** It is recommended to screen iL3s generated from a single group of free-living females (*i.e.,* all females microinjected with the same HDR construct) at the same time so that all iL3s expressing the same fluorophore can be screened using the same filter settings of the microscope.
  c. Pick iL3s with GFP or mScarlet-I expression almost throughout the entire length of the body wall using a paintbrush.
    i. Transfer them to a watch glass containing BU saline.
    ii. Once the paintbrush is dipped in the watch glass containing BU saline, place the paintbrush near the edge of the NGM plate.
    iii. Visually inspect the paintbrush under the microscope to ensure that the iL3 has been transferred to the saline solution and is not still stuck to the paintbrush.
      ***Note:*** Once ∼75–100 iL3s are in the watch glass, it will be difficult to continue depositing iL3s into the dish without inadvertently picking up previously deposited iL3s in the paintbrush. At this point, iL3s can be transferred to a second watch glass containing BU saline; all worms will be pooled in subsequent steps.
  d. Sequentially screen for and pick transgenic iL3s on successive *E. coli* OP50 plates to obtain a total of at least ∼100 iL3s that express GFP and at least ∼100 iL3s that express mScarlet-I across nearly the entire length of the worm.
    ***Note:*** For each fresh *E. coli* OP50 plate, plate iL3s immediately before screening, as described above. It is recommended to minimize the time between plating and screening iL3s, since the worms may crawl up the sides of the plates.
    ***Note:*** Screening of iL3s on the first 1–2 *E. coli* OP50 plates, together with the abovementioned pilot studies, will inform the experimenter whether it is likely that fewer than 100 iL3s that express a fluorophore along 100% of their body length will be isolated. In such cases, iL3s expressing the fluorophore along at least 80% of their body length should also be isolated since some of these iL3s may contain integrated repair cassettes.
  e. Screen iL3s from free-living females microinjected with Microinjection Mix 2 and store them in a separate watch glass, as detailed above.

### Activation of transgenic F_1_ iL3s

**Timing: 40–46 h**

This section describes the steps required for *in vitro* activation^7^^,^^26^^,^^27^ of the screened F_1_ iL3s (Figure 4C).

5. Pool all screened iL3s.
  a. Transfer iL3s from all watch glasses to a single 15 mL conical tube.
    ***Note:*** Because no further steps (prior to inoculation of the gerbil host) require visual screening, this is the step at which iL3s expressing distinct fluorophores can be mixed together.
    **CRITICAL:** It is important to ensure that the watch glasses do not contain leftover iL3s by looking at them under a benchtop microscope. If there are leftover iL3s, wash the watch glasses with small amounts (∼0.5–1 mL) of BU saline and transfer the leftover worms to the conical tube.
  b. Fill the conical tube with BU saline to make a total volume of 10 mL.

6. Axenize iL3s by antibiotic/antifungal treatment (3 h).
  a. Add 100 μL of 100× penicillin/streptomycin, 100 μL of 100× amphotericin B, and 5 μL of 2000× tetracycline hydrochloride to the 10 mL saline suspension containing iL3s.
  b. Mix the contents of the tube by inverting 5–6 times.
  c. Incubate the antibiotic/antifungal-treated worms at 22°C–23°C under dark conditions for 3 h.

***Note:*** To prevent worms from settling at the bottom of the tube, which could lead to hypoxic conditions, it is recommended to place the tube in a horizontal position inside a secondary container (*e.g*., a 15-cm glass Petri dish).

**CRITICAL:** Tetracycline hydrochloride is light-sensitive. Thus, it is critical to incubate the tube under dark conditions by either storing it in the dark or wrapping the tube with aluminum foil to shield it from light.

7. Prepare and pre-incubate activation media (1–3 h).
  a. Aliquot 10 mL Dulbecco’s Modified Eagle Medium (DMEM) into a 15 mL conical tube.
    ***Note:*** A freshly opened bottle of DMEM can be used for 2 weeks after opening when stored at 4°C.
  b. Add 100 μL of 100× penicillin/streptomycin, 100 μL of 100× amphotericin B, and 5 μL of 2000× tetracycline hydrochloride to the DMEM, and then mix by inverting the tube 5–6 times.
  c. Fill the outer perimeter of wells in a lidded 96-well round-bottom plate with 110 μL ddH_2_O, as shown below.
  d. Depending on the number of worms to activate, fill the wells of the plate with 110 μL of antibiotic/antifungal-treated DMEM, as shown in Table 3, and cover the plate with the lid.
    ***Note:*** Each well should contain no more than ∼20–50 worms. For example, if there are 400 worms to activate, fill 8–14 wells of the plate with DMEM.
  e. Line the bottom of a secondary container with wet paper towels and transfer the plate to the container.
  f. Transfer the container to a humidified incubator at 37°C and 5% CO_2_ and incubate for 1–3 h.
    **CRITICAL:** It is important that the antibiotic/antifungal-treated DMEM is pre-incubated for at least 1 h before adding worms. This allows the DMEM to attain a pH of ∼7, which is essential for the survival of worms during activation.

8. Add axenized iL3s to activation media.
  a. After 3 h of axenization, centrifuge the conical tube at ∼3000 × *g* for 2 min.
  b. Gently remove supernatant, leaving ∼1.5 mL liquid containing the worm pellet.
  c. Transfer the iL3s to a microcentrifuge tube.
  d. Centrifuge the tube at 1500–2400 × *g* for 2 min.
  e. Gently pull out 800–900 μL supernatant without disturbing the worm pellet.
  f. Centrifuge the tube for an additional 1 min for a tighter worm pellet.
  g. Gently remove the supernatant without disturbing the worm pellet.
    ***Note:*** To avoid diluting the DMEM in the 96-well plate, you do not want to add more than 5 μL of liquid containing iL3s to each well. Thus, it is important to pre-calculate the final volume of iL3-containing liquid that you will be distributing into the wells. For example, if there are 12 DMEM-filled wells, the final volume of iL3-containing liquid should not exceed 60 μL.
    ***Note:*** Alternatively, steps e-g may be avoided. Instead, the worm pellet can be removed in the desired final volume with a pipette and then transferred to a new microcentrifuge tube. For some experimenters, this results in less worm loss than removing the supernatant (optional).
    **CRITICAL:** It is strongly recommended that you save the supernatant after each centrifugation step to look for and retrieve any non-pelleted worms. These are then placed in the main collection tube.
  h. Transfer no more than 5 μL of liquid containing worms into each well of the pre-incubated 96-well plate.
  i. Incubate the plate with worms in a humidified incubator at 37°C and 5% CO_2_ for 36–42 h.

**Table 3:** Visual representation of how to prepare a 96-well plate for activating iL3s

ddH_2_O	ddH_2_O	ddH_2_O	ddH_2_O	ddH_2_O	ddH_2_O	ddH_2_O	ddH_2_O	ddH_2_O	ddH_2_O	ddH_2_O	ddH_2_O
ddH_2_O											ddH_2_O
ddH_2_O				DMEM	DMEM	DMEM					ddH_2_O
ddH_2_O				DMEM	DMEM	DMEM					ddH_2_O
ddH_2_O				DMEM	DMEM	DMEM					ddH_2_O
ddH_2_O				DMEM	DMEM	DMEM					ddH_2_O
ddH_2_O											ddH_2_O
ddH_2_O	ddH_2_O	ddH_2_O	ddH_2_O	ddH_2_O	ddH_2_O	ddH_2_O	ddH_2_O	ddH_2_O	ddH_2_O	ddH_2_O	ddH_2_O

Additional wells containing DMEM may be necessary, depending on the number of worms you are activating (see below).

### Infection of gerbil hosts with candidate mutant activated iL3s by oral gavage

**Timing: 1–1.5 h**

This section describes the steps required to infect hosts with activated F_1_ iL3s to complete their parasitic life cycle and propagate (Figure 4C).

9. Prepare activated iL3s for infecting hosts.
  a. After 36–42 h of activation, pool all activated iL3s from the 96-well plate into a microcentrifuge tube with a screw cap and O-ring.
  b. Centrifuge activated iL3s at ∼3500 × *g* for 2 min.
  c. Remove supernatant until ∼100 μL liquid is left in the tube.
  d. Repeat the centrifugation process (if necessary) until all activated iL3s are collected from the 96-well plate.
    ***Note:*** To prevent loss of worms, save the supernatant, retrieve any non-pelleted worms, and place them in the main collection tube.
  e. Wash activated iL3s three times with 900 μL of 1× PBS solution.
  f. Resuspend worms in a final volume of 100 μL 1× PBS solution.
    ***Note:*** Use fresh PBS for this step to ensure sterility.

10. Infect hosts with activated iL3s by oral gavage.
  a. Transport the tube with activated iL3s to an ABSL-2 vivarium.
  b. Using a 1 mL Luer-Lok syringe fitted with a sterile oral gavage needle, gently resuspend worms in sterile 1× PBS and fill the syringe with the entire 100 μL dose of transgenic, activated iL3s in sterile 1× PBS.
    **CRITICAL:** Before pulling up worms, backfill the syringe with ∼100 μL air. This will minimize the amount of liquid (and number of worms) left in the syringe after oral gavage.
    ***Note:*** To prevent activated iL3s from sticking to the inside of the syringe, the worms should be drawn up immediately before gavage of the gerbil.
  c. Infect a Mongolian gerbil (host) with activated iL3s by oral gavage.
  d. To minimize the number of leftover worms in the syringe, fill the syringe with an additional 100 μL of fresh sterile 1× PBS solution and gavage a second gerbil with this solution.
  e. Co-house the two gerbils in the same cage. When patent, their feces will be collected together and used to isolate worms.
    ***Note:*** It is recommended that each infection uses a dose of at least 200 activated iL3s, although the dose can range from 100–800 activated iL3s. Doses of fewer than 100 activated iL3s may not cause a patent infection, and doses of >800 activated iL3s may cause illness to the gerbil. Approximately half of the activated iL3s will express one fluorophore (*e.g.,* GFP) from the repair template, while the other half will express the other fluorophore (*e.g.,* mScarlet-I) from the repair template. If excess iL3s expressing each fluorophore are collected during screening, they can be activated and used to infect additional gerbils, which can be co-housed in groups of up to 5 gerbils.

### Isolation and propagation of homozygous knockout iL3s

**Timing: 1–2 months from time of initial gerbil infection**

**Figure 5:**
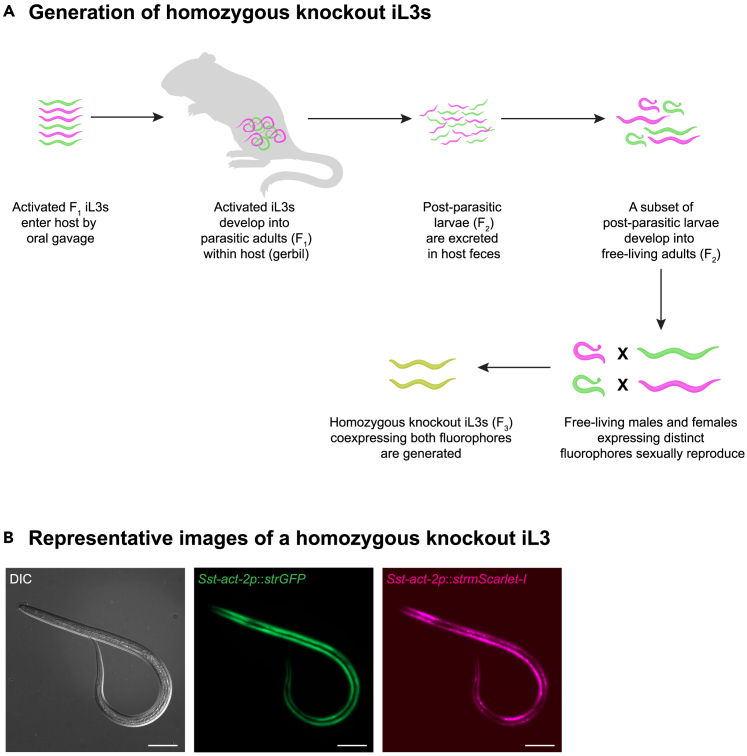
Generation and identification of homozygous knockout iL3s (A) Schematic showing the generation of homozygous knockout F_3_ iL3s in the context of the *S. stercoralis* life cycle. Pink worms represent worms expressing mScarlet-I; green worms represent worms expressing GFP; yellow worms represent worms expressing both mScarlet-I and GFP. (B) Representative images of an iL3 containing a homozygous disruption of the *Sst-gcy-9* gene. The iL3 co-expresses mScarlet-I and GFP in body-wall muscle, with one fluorophore expressed from each integrated HDR construct. Head is to the left. DIC = differential interference contrast microscopy; “*str*” = *Strongyloides*-codon-optimized. Images were acquired on a Zeiss AxioObserver 7 microscope using Zeiss Zen 3.3 software (blue edition) and were processed using FIJI.^22^ Images were acquired as z-stacks and maximum intensity projections are shown. Scale bar = 50 μm.

This section describes the steps required to isolate homozygous knockout iL3s in the F_3_ generation and propagate the stable knockout line through successive generations (Figures 4D and 4E). The activated F_1_ iL3s that were oral-gavaged into the gerbil will develop into parasitic adults within the gerbil. The parasitic adults reproduce by parthenogenesis and their progeny (post-parasitic young larvae) are excreted in host feces, where a subset of them will develop into free-living adult males and females (F_2_). The free-living adults will reproduce sexually to generate F_3_ iL3s. When free-living males and females (F_2_) containing distinct HDR cassettes in complementary alleles of the gene mate and reproduce, a subset of the resulting iL3 progeny (F_3_) will contain a homozygous disruption of both alleles and thus will co-express two fluorophores in their body-wall muscles (*i.e.,* they will be red/green dual-colored iL3s) (Figures 5A and 5B).

11. Isolate iL3s from host feces.
  a. After at least 14 days post-oral-gavage (when the host starts shedding post-parasitic young larvae in feces), collect feces from the host and prepare fecal-charcoal plates as described above.
    ***Note:*** Multiple days of collections will likely be needed to obtain sufficient numbers of F_3_ iL3s for the next round of infection. *S. stercoralis* infections in gerbils peak at around 23 days post-infection. Thus, it may be helpful to collect feces around day 23 post-infection to obtain larger numbers of iL3s.
  b. Incubate the fecal-charcoal plates at 23°C for 7–10 days to allow the post-parasitic young larvae to develop into iL3s.
  c. Collect iL3s using a Baermann apparatus, as described above.
    ***Note:*** Wait 2–3 h before collecting iL3s from the Baermann apparatus to maximize worm output.
  d. Store iL3s in BU saline in a microcentrifuge tube until screening.
  e. Alternatively, collect iL3s 1 h after setting up the Baermann apparatus and start screening them. If more iL3s are needed after screening the first batch, collect more from the Baermann apparatus (optional).

12. Screen for homozygous knockout iL3s.
  a. Depending on the density, transfer iL3s in 2–10 μL liquid droplets to an *E. coli* OP50 plate.
  b. After the liquid droplet dries and iL3s disperse across the plate, examine the plate under a fluorescence dissection microscope to identify iL3s that co-express two distinct fluorophores (*e.g.*, GFP and mScarlet-I) in their body-wall muscle (Figure 5B).
  c. Pick single iL3s co-expressing both fluorophores using a paintbrush and transfer them to watch glasses containing ∼1 mL BU saline, such that each watch glass contains no more than ∼75–100 iL3s, as described above.

***Note:*** Aim to pick approximately 200 dual-colored iL3s into 2–3 watch glasses. These iL3s can be pooled, activated, and then used to infect a single gerbil (with a second co-housed gerbil receiving the wash from the oral gavage needle, as described above).

**CRITICAL:** It is critical to ensure that only the iL3s co-expressing both fluorophores are isolated. This will ensure 100% homozygosity in successive generations and establish a stable knockout line. It is important to transfer iL3s onto the *E. coli* OP50 plate at relatively low densities such that iL3s of other categories (non-transgenic iL3s or iL3s expressing a single fluorophore) are not accidentally picked up by the paintbrush.

***Note:*** It is generally recommended to pick a total of ∼200 dual-colored iL3s, since higher numbers may increase the chance of accidentally picking non-homozygous worms. In our experience, an inoculum of 200 activated iL3s is sufficient to establish a patent infection in most cases. However, if there is reason to suspect that the mutation of interest may render the iL3s less capable of establishing an infection, a larger number of dual-colored activated iL3s (*e.g.,* ∼500) can be used to infect a gerbil. In all cases, it is recommended to use higher numbers of a mixed population of transgenic F_2_/F_3_ activated iL3s for inoculation of a separate gerbil in parallel (with an additional co-housed gerbil receiving the wash from the oral gavage needle, as described above), which can serve as a backup in case the infection with homozygous knockout activated iL3s does not take. Note that these backup gerbils must be housed separately from the gerbils that receive the dual-colored activated iL3s. If the initial infection with homozygous activated iL3s does not take, worms from the backup gerbils can be used to try again to establish a homozygous knockout line. In this case, up to 800 dual-colored activated iL3s can be used to try to establish a homozygous knockout line.

***Note:*** If dual-colored F_3_ iL3s cannot be detected while screening, it is recommended to set up crosses between F_2_ L4-stage larvae or free-living adults. For these crosses, isolate transgenic F_2_ free-living adults from fecal-charcoal plates that were incubated at 25°C for 1 day using a Baermann apparatus. Plate all the isolated free-living adults onto an *E. coli* OP50 plate. Next, under a fluorescence dissection microscope, pick unmated free-living females of one color (*e.g.,* expressing mScarlet-I) and free-living males of the other color (*e.g.*, expressing GFP) using a paintbrush and transfer them onto a fresh *E. coli* OP50 plate. To ensure that the free-living females have not previously mated, L4 female larvae can be used in these crosses. Allow mating piles to form on the *E. coli* OP50 plate. Approximately 1 h after the mating piles have formed, flood the *E. coli* OP50 plate with water and serially transfer all the worms onto a freshly made fecal-charcoal plate, as detailed above. Use a similar procedure to set up crosses between free-living females that express GFP and free-living males that express mScarlet-I; these crosses should be plated on a distinct fecal-charcoal plate. Approximately 5 days later, isolate iL3s from both plates using a Baermann apparatus and screen for worms expressing both GFP and mScarlet-I.

13. Activate screened iL3s for 36–42 h, as described above.
14. Pool activated iL3s and infect one or more gerbils by oral gavage, as described above.
15. Repeat the process for continued propagation of the stable line.

***Note:*** Once the stable line is established, it is recommended to infect two or more gerbils, each with ∼500 activated iL3s, for strain maintenance. These gerbils can be co-housed in the same cage (up to 5 gerbils per cage) and their feces collected together. Homozygous mutant iL3s or worms of any other life stage can now be tested for a mutant phenotype.

***Note:*** Since the host is infected with F_3_ activated iL3s specifically co-expressing GFP and mScarlet, all resulting post-parasitic larvae that are excreted in host feces should be homozygous for the disruption of the targeted gene locus. As a result of allelic segregation, ∼50% of the F_5_ iL3s that develop from these post-parasitic larvae will co-express both fluorophores, ∼25% will express GFP only, and ∼25% will express mScarlet-I only. At this point, all the iL3s, including the red-only iL3s and the green-only iL3s, will have homozygous gene disruptions. The red-only or green-only iL3s can be isolated, activated, and oral gavaged into gerbils for establishing all-red or all-green lines, as needed.^1^

***Note:*** At this stage, for maintaining the stable line by successive propagation through hosts, the screening step may be avoided. However, before each round of infection, it is important to visually confirm that the entire population of activated iL3s are transgenic and express either one or both fluorophores in their body-wall muscle. If any non-fluorescent activated iL3s are identified (because the non-fluorescent iL3s were accidentally carried over in a previous step), the line is not yet homozygous, and another round of red/green selection is needed to establish a homozygous line. In addition, it is generally recommended to confirm that the iL3s used for host passage include both red and green iL3s, or dual-colored iL3s, so that both fluorophores are maintained. This allows maximum flexibility for generating all-red or all-green lines in the future.

***Note:*** Once the stable knockout line is established, the experimenter can attempt to propagate the line by oral gavage of gerbils with non-activated mutant iL3s. Use of non-activated iL3s improves efficiency by omitting the time needed for activation. In this case, collect iL3s from fecal-charcoal plates using a Baermann apparatus, as described above. Wash the iL3s three times in sterile 1× PBS. Resuspend the iL3s in sterile 1× PBS and oral gavage into gerbils, as described above. We do not recommend orally gavaging non-activated iL3s if these iL3s were isolated by screening on *E. coli* OP50 plates. Additionally, if attempting to propagate the stable knockout line by using non-activated iL3s for the first time, it is highly recommended to establish a mixed population line in parallel, as described below.

**CRITICAL:** In addition to collecting F_3_ iL3s co-expressing both fluorophores to establish a homozygous knockout line, it is strongly recommended to also establish a mixed population line in parallel. This mixed population line can be used as a backup in case the homozygous mutants fail to infect a host. To establish a mixed population line, collect a mixed population consisting of F_2_/F_3_ iL3s expressing GFP only and F_2_/F_3_ iL3s expressing mScarlet-I only in their body-wall muscle across most of their body length; this mixed population of iL3s will likely consist of both homozygous and heterozygous mutants. Pool all iL3s expressing a single fluorophore and infect a host. This strategy allows maintenance of the line even if homozygous mutants are unable to establish an infection. However, if maintaining a mixed population line, it is necessary to confirm that your iL3 population consists of both GFP-positive and mScarlet-I-positive iL3s every generation before host passage. It is also necessary to actively screen for and select GFP-positive iL3s and mScarlet-I-positive iL3s every 2–3 generations to ensure continued propagation of both GFP-positive and mScarlet-I positive iL3s carrying the mutant allele.

### Genotyping worms to confirm homozygous disruption of the genetic loci

**Timing: 5–6 h**

***Note:*** While the dual-selection marker approach is a reliable visual indication of homozygous gene disruption, it is strongly recommended to confirm the generation of homozygous mutants by genotyping a subset of F_3_ iL3s co-expressing both fluorophores. It is also recommended to genotype iL3s (or other life stages) of later generations to confirm the establishment of a stable homozygous knockout line.

**Figure 6:**
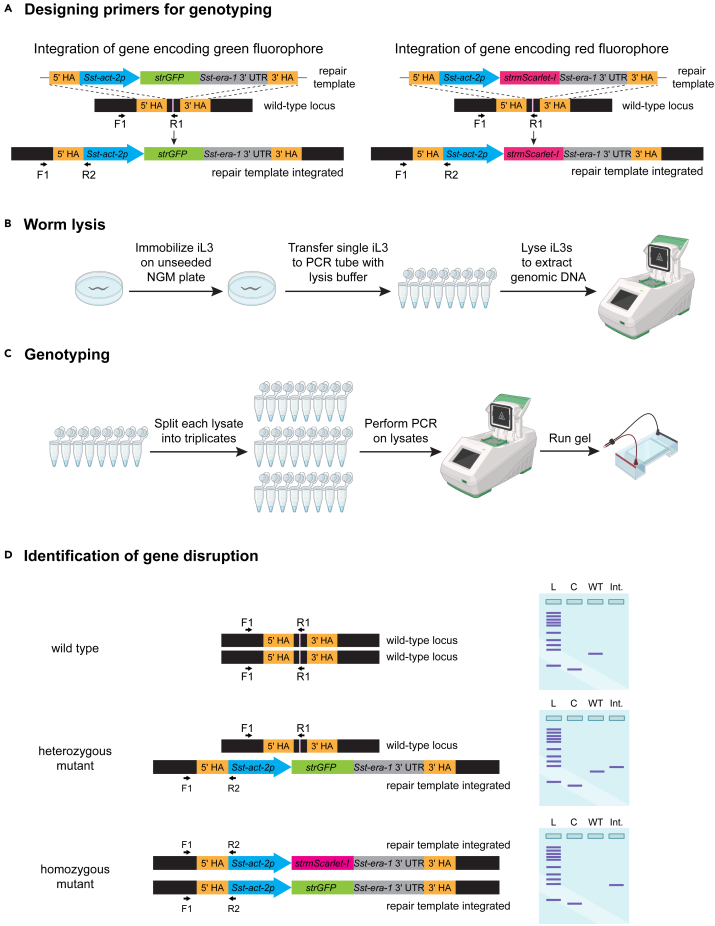
Confirmation of homozygous gene disruption by PCR-genotyping (A) Three different combinations of primer pairs are required. The first pair amplifies a part of the first exon of the *Sst-act-2* gene to confirm the presence of genomic DNA^7^ (not shown). A second pair (F1 and R1) detects the wild-type locus near or overlapping the CRISPR site. A third pair (F1 and R2) detects integration of the repair cassettes. The CRISPR target site is indicated in light pink. (B–D) Steps involved in the lysis and PCR-genotyping of single iL3s to confirm gene disruption. For the gel schematics, L = 1 kb ladder; C = positive control PCR reaction that tests for the presence of genomic DNA (reaction amplifies part of exon 1 of the *Sst-act-2* gene); WT = PCR reaction that only shows an amplification product if the wild-type locus remains intact on at least one chromosome; Int. = PCR reaction that only shows an amplification product if the repair template has successfully integrated at the target locus.

This section describes the steps required to genotype iL3s for confirming homozygous disruption of the gene of interest (Figure 6).

16. Design primer sets for genotyping iL3s (Figure 6A; Table 4).
  a. The first primer pair should amplify exon 1 of the endogenous *S. stercoralis act-2* (*Sst-act-2*) gene as a positive control for the presence of gDNA in each PCR reaction.
  b. The second primer pair should amplify the wild-type locus near the CRISPR target site.
  c. The third primer pair should flank either the 5′ or 3′ homology arm and detect integration of the HDR cassette.
17. Prepare genomic DNA by lysing worms (Figure 6B).
  a. To 167 μL of worm lysis buffer, add 2 μL of proteinase K and 3 μL of 2-mercaptoethanol to prepare worm lysis solution.
  b. Transfer 6 μL of worm lysis solution to PCR tubes.
    ***Note:*** It is recommended to genotype at least 16–24 iL3s of each genotype (wild type and knockout) in separate PCR tubes.
  c. On an unseeded NGM plate, transfer single iL3s in droplets and immobilize them by adding 1% nicotine solution.
  d. After the droplets dry and the iL3s are paralyzed, pipet out ∼1 μL of worm lysis solution from a PCR tube and transfer a single iL3 into the same PCR tube via the lysis solution.
    ***Note:*** iL3s tend to stick to the plastic surfaces of pipet tips. It is recommended to check the lysis solution in the tube under a microscope to confirm the presence of the worm. If the worm is not transferred, pipet up and down several times until the worm gets into the lysis solution.
  e. After transferring individual iL3s to the PCR tubes, store the tubes at −80°C for at least 20 min prior to lysis.
    **Pause point:** The worms can be stored at −80°C for days or even months prior to lysis.
    ***Note:*** Not all iL3s need to be lysed and genotyped on the same day.
  f. For lysis, transfer the PCR tubes to a thermocycler and incubate at 65°C for 2 h followed by 95°C for 15 min.
  g. Cool down the lysis reaction at 4°C.

18. Genotype single iL3s (Figure 6C).
  a. For each genotype, split each worm lysate into triplicates in PCR tubes with ∼2 μL lysate in each tube.
  b. Prepare three different PCR reaction mixes (one for each group of lysates) containing different primer pairs, as described in Table 4.
  c. For a given primer pair, set up a 50 μL PCR reaction for each iL3 lysate, as described in Table 5.
  d. For all PCR reactions, use the thermocycler conditions shown in Table 6.
    ***Note:*** The recommended annealing temperature will vary depending on the melting temperature of the primers and the extension time may vary depending on the size of the amplicons.
  e. Load 15–25 μL of each PCR reaction per well of a 1%–2% agarose gel and run gel at ∼120 V for 40–60 min in 1× TBE buffer.
  f. Image the gel and save the image for documentation.
    ***Note:*** At this stage, we recommend confirming the identity of the amplicons from PCR reactions for detecting the wild-type locus and the integration site by sending them for sequencing.

**Table 4:** Primer combinations for each PCR reaction mix

PCR reaction	Control primers		Gene-specific primers		Function
	Forward	Reverse	Forward	Reverse	
1	C1	C2			Amplify exon 1 of the endogenous *Sst-act-2* gene to detect presence of genomic DNA
2			F1	R1	Detect wild-type locus around CRISPR target site
3			F1	R2	Detect integration of repair cassette

The binding sites for the primers for PCR reaction mixes 2 and 3 are shown in Figure 6A.

**Table 5:** PCR reaction mix for PCR-genotyping single iL3s

Reagent	Amount
Worm lysate (DNA template)	2 μL
10× Platinum Taq Buffer	5 μL
10 mM dNTP mix	1 μL
50 mM MgSO_4_	2 μL
10 mM Forward primer	1 μL
10 mM Reverse primer	1 μL
Platinum Taq enzyme	0.5 μL
ddH_2_O	37.5 μL

**Table 6:** PCR cycling conditions for PCR-genotyping single iL3s

Steps	Temperature	Time	Cycles
Initial Denaturation	94°C	2 min	1
Denaturation	94°C	15 s	34 cycles
Annealing	53°C	30 s	
Extension	68°C	1 min	
Final extension	68°C	5 min	1
Hold	4°C	forever	

## Expected outcomes

This protocol is used to generate stable knockout strains in *S. stercoralis.* Using CRISPR combined with a dual-marker selection approach, this protocol enables the generation of mutant lines and their perpetual maintenance by host passage. In our original paper, we disrupted the receptor guanylate cyclase gene *Sst-gcy-9* in *S. stercoralis* and generated stable homozygous mutants.^1^ We observed defects in CO_2_-evoked behavioral responses in *Sst-gcy-9* homozygous mutant worms. We also observed that the *Sst-*BAG sensory neurons were unable to detect CO_2_ in the *Sst-gcy-9* mutants.^1^ However, this protocol can be used to study most genes of interest in *S. stercoralis* (for limitations, see below).

## Limitations

Although this protocol can be used to knock out many genes of interest in *S. stercoralis*, it cannot be used to generate stable homozygous mutant lines for genes that are essential for survival or essential for infecting a host. Such genes may include those required for embryonic development, development of a specific life stage (*e.g.*, iL3s or parasitic adults), activation of iL3s, and egg laying by parasitic adults. Moreover, for genes that are expressed in multiple cells or tissues, or at multiple stages of the life cycle, this method cannot be used to tease apart their roles in specific cells or at specific life cycle stages. To overcome these limitations, alternate approaches that enable spatiotemporal control of gene disruption, such as the Cre/Lox, cGal4-UAS, Q-, and FLP/FRT systems,^28^^,^^29^^,^^30^^,^^31^^,^^32^^,^^33^ may be considered. However, these systems have not yet been applied to *S. stercoralis*.

## Troubleshooting

### Problem 1

Absence of F_3_ iL3s co-expressing both fluorophores. See step-by-step method details – step 12.

### Potential solution

• Prepare fecal-charcoal plates across multiple days of patency (days 14–44 post-infection). Incubate the plates at 23°C for 7–10 days and screen for iL3s expressing both fluorophores from each plate. In such cases, it is recommended to collect feces from hosts across multiple days around the peak of the infection (around day 23) to maximize post-parasitic larval output.
• If the problem persists and F_3_ iL3s expressing only one of the two fluorophores are found, it is possible that the other HDR cassette did not get integrated into the genome. In *S. stercoralis*, only integrated transgenes are expressed past the F_1_ generation.^5^^,^^7^^,^^25^^,^^34^^,^^35^ In this case, microinjecting more free-living P_0_ adult females to generate more transgenic progeny may solve the problem (see below).
• Before attempting to generate a stable line, it is important to run pilot experiments (as suggested above) and verify that both repair cassettes are integrating in sufficient numbers of F_1_ iL3s. This can be done by isolating F_1_ iL3s expressing the fluorophore in >80% of their body-wall muscle and genotyping single iL3s to determine the percentage of iL3s with integrated repair cassettes. Of note, integration of the repair cassette can be heterozygous and/or mosaic in the F_1_ generation, which may result in evidence of both an intact wild-type locus and integration upon genotyping. For this reason, it is more important in the F_1_ generation to assess for the presence of integration than to identify homozygous mutants. Because of mosaicism, the absence of homozygous mutants in the F_1_ generation upon genotyping does not mean that it will be impossible to generate homozygous mutants in the F_3_ generation. If there is a low percentage of F_1_ iL3s containing integration of a specific repair cassette, perhaps because of an inefficient CRISPR target site, microinject more free-living adults (P_0_) with the microinjection mix containing that HDR construct to increase the number of desired iL3s and repeat the stable line attempt. See step-by-step method details – step 2.
• If you obtain the expected number of F_3_ iL3s singly expressing each of the two fluorophores but you do not obtain iL3s that co-express both fluorophores, it is possible that homozygous mutants are non-viable or undergo developmental arrest before the iL3 stage. In this case, a mixed population line can be maintained. To maintain a mixed population line, collect F_2_/F_3_ iL3s singly expressing each fluorophore, pool them, and propagate them by host passage, as described above. If the gene is not completely recessive, the heterozygous mutants may exhibit a partial phenotype.

### Problem 2

Low numbers of F_3_ iL3s co-expressing both fluorophores (homozygous mutants). See step-by-step method details – step 12.

### Potential solution

• Collect F_3_ iL3s that express a single fluorophore of each color in addition to the iL3s that co-express both fluorophores. Pool all iL3s and infect a host. This will amplify the number of iL3s co-expressing both fluorophores in the F_5_ generation. Note that to establish a stable homozygous knockout line, it will be necessary to screen the F_5_ generation for dual-colored iL3s and select only dual-colored iL3s for reinfecting the next gerbil.
• Alternatively, collect F_2_ free-living adults that express a single fluorophore and set up crosses between unmated free-living females of one color and free-living males of the other color. Recover the crosses on fecal-charcoal plates and screen for dual-colored F_3_ iL3s, as described above. To ensure that the free-living females have not previously mated, L4 female larvae can be used in these crosses.

### Problem 3

No worms or low numbers of worms in the F_4_/F_5_ generation when homozygous dual-colored F_3_ activated iL3s were used as an inoculum. See step-by-step method details – step 15.

### Potential solution

This problem can arise if the gerbil infection did not take, which can happen occasionally by chance. It can also happen if none or only a small subset of the homozygous knockout F_3_ iL3s can complete their life cycle inside the gerbil, or if the homozygous knockout F_3_ parasitic adults have reproductive defects. Moreover, this problem can also arise if the homozygous F_4_ post-parasitic larvae have developmental defects or the F_4_ free-living adults have mating defects, resulting in the absence of or low numbers of F_5_ iL3s.

• If there are low numbers of worms, collect F_4_/F_5_ iL3s across multiple days post-infection and infect a host with a greater number of activated iL3s. Infect multiple gerbils if enough transgenic iL3s are collected. Up to 800 iL3s can be used to infect a single gerbil via oral gavage without causing illness.
• If there are no worms, it is possible the gerbil infection did not take. It is usually worth repeating the protocol a second time to see if the next gerbil infection is successful. Alternatively, if a mixed population line was established in parallel with the homozygous knockout line, isolate dual-colored F_5_ iL3s from the mixed population line. Use these iL3s to infect the next gerbil and determine whether this round of infection is successful. If the process is repeated using either approach but there are still no worms shed from the host when homozygotes are used to start the infection, the line will likely need to be maintained as a mixed population, as described above.

***Note:*** Even if homozygous knockout iL3s cannot be obtained directly in the post-parasitic generation due to their inability to infect a host, it may still be possible to obtain homozygous knockout iL3s in the post-free-living generation by crossing transgenic free-living males and females from the mixed population line, as described above.

### Problem 4

Presence of non-transgenic iL3s in the F_4_/F_5_ generation. See step-by-step method details – step 15.

### Potential solution

This suggests that a stable homozygous knockout line has not been established yet. This may happen if non-transgenic iL3s or iL3s expressing a single fluorophore were accidentally isolated in addition to iL3s co-expressing both fluorophores during the screening of F_3_ iL3s.

• Collect F_5_ iL3s that co-express both fluorophores exclusively and use them to infect a host. It may be helpful to transfer fewer iL3s onto the screening plate to prevent overcrowding and unintentional carry-over of non-transgenic iL3s or iL3s expressing a single fluorophore.

***Optional:*** To minimize the chance of unintentional carry-over of non-transgenic iL3s, the experimenter may choose to set up crosses between unmated free-living females and free-living males that co-express both fluorophores (or, if the gene of interest is on the X chromosome, unmated free-living females that co-express both fluorophores and free-living males that express a single fluorophore), as described above. All of the iL3 progeny resulting from these crosses should be homozygous mutants (expressing either a single fluorophore or both fluorophores).

### Problem 5

No phenotype or partial phenotype in homozygous mutants. See step-by-step method details – step 15.

### Potential solution

This problem could arise due to incomplete disruption of the gene. For example, if critical functional domains are not disrupted, it may result in a lack of phenotype or a partial phenotype.

• Identify a CRISPR cut site within a predicted critical functional domain of the gene and redesign the CRISPR components.
• It may be helpful to identify two CRISPR cut sites that are far enough apart to result in a large deletion of the gene locus. This may eliminate large functional domains or multiple domains required to drive protein function.^13^

The lack of a phenotype or a partial phenotype may also arise due to complete or partial genetic redundancy, where multiple genes contribute to the same phenotype.

• If two or more genes of the same family are in tandem on the same chromosome, using two CRISPR sites spanning a large segment of the multi-gene locus may be useful. This would disrupt multiple genes simultaneously and may address the problem if the genes are functionally redundant.
• If the redundant genes are not in proximity or are located on distinct chromosomes, generating double mutants by disrupting two genes in the same strain may address the problem. To disrupt two genes in the same animal, first generate a red/green single knockout line of the first gene of interest. From this line, select red-only or green-only iL3s for passage through a gerbil to generate a red-only or green-only homozygous knockout line. Next, microinject the CRISPR constructs for the second gene of interest into free-living females from the single-color line, using HDR constructs that contain two different colors. For example, if you start with a red-only single knockout line, you can microinject CRISPR constructs where HDR #1 contains a *strGFP* cassette and HDR #2 contains a *strElectra2::*P2A*::strElectra2* cassette. The protocol above can then be repeated using green/blue color selection on the all-red line such that you end up with double-knockout worms that are red, green, and blue. From this stable line, some worms will be red and green only, some will be red and blue only, and some will be red, green, and blue.

***Note:*** This approach can only be used to create double knockouts. To disrupt more than two genes in the same worm, it is necessary to use at least two distinct promoters with different expression patterns in the HDR constructs so that successful targeting at the different loci can be identified by visual inspection.

## Resource availability

### Lead contact

Further information and requests for resources and reagents should be directed to and will be fulfilled by the lead contact, Elissa A. Hallem (ehallem@ucla.edu).

### Technical contact

Technical questions on executing this protocol should be directed to and will be addressed by the technical contacts, Navonil Banerjee (nbanerjee1@ucla.edu), Breanna Walsh (breannawalsh@ucla.edu), and Ruhi Patel (ruhiali@ucla.edu).

### Materials availability

This protocol did not generate any new strains or reagents. Strains and reagents used in this protocol are available from the lead contact on request.

### Data and code availability

This protocol did not generate any new data or code.

## Acknowledgments

We thank Matthew Moser and Julian Wagner for providing helpful comments and feedback. We thank Yanying Dai for gerbil husbandry, gerbil oral gavage, and fecal collections. We thank Tiffany Mao for making solutions and media used in this protocol and assisting with the key resources table. This work was funded by National Institutes of Health F32AI147617 and National Institutes of Health T32AI007323 (PI: P. Johnson) to N.B.; National Institutes of Health T32GM152342 (PIs: D. Dawson and O. Ajijola), a Lothar-Anne Rosenthal Medical Scientist Training Program Fellowship, a UCLA Molecular Biology Institute Whitcome Fellowship, and National Institutes of Health F30AI179222 to B.W.; National Institutes of Health F32AI174816 to R.P.; and National Institutes of Health R01DC021489 to E.A.H. Figures were created using BioRender.com.

## Author contributions

Conceptualization and methodology, N.B., B.W., R.P., M.L.C., and E.A.H.; investigation, N.B., B.W., R.P., and M.L.C.; writing – original draft, N.B.; writing – review and editing, N.B., B.W., R.P., M.L.C., and E.A.H.; funding acquisition, resources, and supervision, E.A.H.

## Declaration of interests

The authors declare no competing interests.
